# A Newly-Discovered Mutation in the RFX6 Gene of the Rare Mitchell-Riley Syndrome

**DOI:** 10.4274/jcrpe.2387

**Published:** 2016-06-06

**Authors:** Nusrat Khan, Waleed Dandan, Noura Al Hassani, Suha Hadi

**Affiliations:** 1 Tawam Hospital, Clinic of Pediatrics and Neonatology, Al Ain, United Arab Emirates; 2 Tawam Hospital, Clinic of Pediatrics and Endocrinology, Al Ain, United Arab Emirates

**Keywords:** Mitchell-Riley syndrome, Diabetes, pancreatic hypoplasia

## Abstract

Mitchell-Riley syndrome is a genetic disorder characterized by neonatal diabetes, pancreatic hypoplasia, intestinal atresia and/or malrotation, biliary atresia, and gallbladder aplasia or hypoplasia. It was considered a variant of the Martinez-Frias syndrome with similar phenotypic characteristics, except for neonatal diabetes and tracheoesophageal fistula. However, the genetic mutation in (regulatory factor X on chromosome 6) RFX6 was only detected in babies who had diabetes, making it different from the previously known mutations for the disease. This is the first reported case of a classical Mitchell-Riley syndrome in the Arab peninsula along with additional features and novel mutations in the RFX6 gene.

## WHAT IS ALREADY KNOWN ON THIS TOPIC?

The Mitchell Riley Syndrome is a recently diagnosed genetic disorder characterised by neonatal diabetes, pancreatic hypoplasia, intestinal atresia, malrotation, biliary atresia, and gallbladder aplasia or hypoplasia. A novel genetic mutation in the RFX6 gene (regulatory factor X on chromosome 6) was detected in babies with neonatal diabetes.

## WHAT THIS STUDY ADDS?

We report a case with neonatal diabetes, pancreatic hypoplasia gall bladder agenesis, duodenal atresia, haemochromatosis, hypospadias and intrauterine growth restriction with some additional features along with a different mutation in the RFX6 gene which has not been reported before.

## INTRODUCTION

The Mitchell-Riley syndrome (1) is a recently diagnosed genetic disorder characterized by neonatal diabetes, pancreatic hypoplasia, intestinal atresia and/or malrotation, biliary atresia, and gallbladder aplasia or hypoplasia (2). It was initially considered as a variant of Martinez-Frias syndrome, diagnosed in 1992, with similar phenotypic characteristics (3). However, the two syndromes differ in that neonatal diabetes is present in Mitchell-Riley, while tracheoesophageal fistula is found in the Martinez-Frias syndrome (4). Over the years, many cases were reported with the same phenotypes, but additional features have also been discovered such as haemochromatosis, thyroid dysfunction, auditory canal defects, hypospadias in males and anteriorly-placed anus in females (5,6). Infants with neonatal diabetes have also been investigated for gene defects for diabetes, such as PLAGL-1 (ZAC), glucokinase and PDX-1 (IPF-1) genes, with negative results (6). In 2010, Smith et al (7) detected a novel genetic mutation in the RFX6 gene (regulatory factor X on chromosome 6) in 6 babies, all of whom had neonatal diabetes. This defect could not be found in babies who had same phenotypic features but did not have neonatal diabetes (8). After that, two further cases were reported, an Israeli Arab patient reported by Spiegel et al (9) and another patient from a Vietnamese family in 2014 reported by Concepcion et al (10). Sansbury et al (11) studied a Turkish family in which 2 double first cousins had intestinal atresia consistent with a diagnosis of Mitchell-Riley syndrome, but did not develop diabetes until the ages of 3 years and 6 years.

Here, we report a case with neonatal diabetes, pancreatic hypoplasia, gall bladder agenesis, duodenal atresia, haemochromatosis, hypospadias, and intrauterine growth retardation (IUGR) with some additional features along with a different mutation in the RFX6 gene which has not been reported before.

## CASE REPORT

This male baby was born, at term, by normal delivery to a consanguineous (third degree) couple from the United Arabian Emirates (UAE) with a prenatal diagnosis of duodenal atresia. The infant had severe IUGR with a birth weight of 1.3 kilograms and practically no subcutaneous fat. Hypospadias was also present. No facial dysmorphism was noted. During the first week of life, the patient developed hyperbilirubinemia with mildly elevated liver enzymes, hyperammonemia, and hyperglycaemia which required insulin. He also tested positive for the direct agglutination test (DAT positive), had renal dysfunction, microangiopathic haemolytic anaemia, and coagulopathy with prolonged activated partial thromboplastin time (APTT). With these problems, he was initially diagnosed and managed as a patient with an inborn error of metabolism. Although the surgery for duodenal atresia was delayed because of thrombocytopenia and severe coagulopathy, these issues were later found to be related to the factor IX deficiency. At the same time, the Doppler test showed a congenital large portosystemic shunt in the liver with a ratio less than 30%. An echocardiogram revealed multiple echogenic masses in the ventricles which were consistent with cardiac rhabdomyoma. An ultrasound of the brain revealed periventricular calcification. TORCH infections and tuberous sclerosis were ruled out by viral and genetic studies.

Once the clotting profile and anaemia had been stabilised by multiple transfusions of fresh frozen plasma (FFP), cryoprecipitate, packed red-blood cells (PRBC), platelets and Factor IX, a laparotomy was performed for duodenal atresia and for a duodenostomy when the baby was 4 weeks of age. The surgery also revealed absence of the gall bladder. A liver biopsy and an endoscopic retrograde cholangiopancreatography (ERCP) could not be performed due to the patient’s poor condition. He had developed severe peritonitis and sepsis, requiring antibiotic and ventilator support for a prolonged period of time. Later on, a hepatobiliary scan (HIDA) revealed poor uptake by the liver and delayed excretion of bile with no visualisation of the gall bladder.

The patient had persistent hyperglycemia, requiring insulin in high doses, followed by hypoglycaemia at intervals. These findings were labelled as neonatal diabetes. Thyroid function tests were slightly abnormal on multiple occasions so a small dose of levothyroxine was started. The ferritin level was also checked and was found to be >6000 µg/L, suggesting hemochromatosis. At the age of nine weeks, severe hypertension developed, which was controlled by antihypertensive medication. Renal Doppler and renal functions were normal at this time.

The baby started tolerating small amounts of expressed breast milk (EBM) two weeks after the surgery. Ingested amounts increased slowly, but soon, he started passing loose, sticky and green-coloured stool. The work-up for malabsorption showed minute levels of stool elastase (<50 µg/g of stool) and low serum lipase which was consistent with severe exocrine insufficiency of the pancreas and possible pancreatic hypoplasia.

Magnetic resonance imaging (MRI) of the abdomen confirmed aplasia of the gall bladder and hypoplasia of pancreas, while the brain MRI showed periventricular calcification. The genetic studies done for Mitchell-Riley syndrome (RFX6 gene locus) confirmed presence of a homozygous mutation in the RFX6 gene (c.1153C>T p.Arg385*), a previously unreported homozygous mutation in exon 11 of the RFX6 gene. Therefore, this was a confirmed case of Mitchell-Riley syndrome with additional features. The parents were not studied for a carrier state.

## DISCUSSION

This is the second case of Mitchell-Riley syndrome diagnosed in a population of Arab ethnicity, the first case ever reported from the Arab peninsula and the ninth case overall (9,10). Although this infant had the classical features of the Mitchell-Riley syndrome including neonatal diabetes, pancreatic hypoplasia, duodenal atresia, gall bladder aplasia, he did not have malrotation or biliary atresia. The infant also had chronic diarrhoea/malabsorption due to severe exocrine pancreatic insufficiency and cholestatic jaundice, findings which have been reported in most of the published cases (5,6,9,10,12). He even had hemochromatosis, reported only by Martinovici et al (5). However, similar to other patients, this infant also had several features overlapping with the Martinez-Frias syndrome such as hypothyroidism (4,12,13,14), severe IUGR, and hypospadias (4,15). In most of the previously known patients, severe hypoplasia or aplasia of the gall bladder and biliary atresia with acholia were the main features and the Kasai procedure was successfully carried out in one of these patients (2,10,12,13,14). Although the gall bladder could not be visualized on the HIDA scan, in the MRI scan nor per-operatively, our patient never had acholic stools. He had mild direct hyperbilirubinemia and elevated liver enzymes and unfortunately, we could not carry out an ERCP or a liver biopsy to confirm a diagnosis of biliary atresia. Our patient also had anaemia during his first week and tested DAT positive; since he had thrombocytopenia, microangiopathic anaemia was considered. Anaemia was also found in one of the previously reported cases and a blood transfusion was given. However, a cause for anaemia was not mentioned and thrombocytopenia was never reported in any patient (10).

This patient had various other previously unreported features such as cerebral calcification and cardiac rhabdomyomas. A cardiac lesion was reported previously, but that patient had a septal defect. Cerebral lesions were most likely never detected, due to most of the infants dying in their first few days of life with no reports of post-mortem findings. Our patient had coagulopathy with factor IX deficiency which has also not been reported in any other patient. Gastrointestinal bleeding was noted in some patients, but the cause had not been identified. The portosystemic shunt and hyperammonemia in our patient can be considered an incidental finding or possibly a developmental defect along with the intestinal atresia. The shunt in our baby closed spontaneously and the ammonia level returned to normal. A minimal shunt was shown afterwards. The systemic hypertension requiring antihypertensive medicine was another finding not reported before and unfortunately, its cause could not be identified; either it was a part of the syndrome due to the distinctive genetic mutation or just an additional finding. The genetic mutation was also different from previously reported patterns, which consisted of c.649T4C homozygous (6), c.781-2_787delAGGTT-GATAinsG homozygous (9) and c.779A4C homozygous (10), while this patient had a previously unreported mutation: c.1153C>T p.Arg385*.

In conclusion, we have reported a confirmed case of Mitchell-Riley syndrome with a previously unreported homozygous mutation in exon 11 of the RFX6 gene. To our knowledge, this is the first ever reported case from the Arab peninsula. Furthermore, the patient had atypical additional features which could be related to a new mutation which needs to be further explored.

**Ethics**

Informed Consent: It was taken.

Peer-review: External and Internal peer-reviewed.

## AUTHORSHIP CONTRIBUTIONS

Concept: Nusrat Khan, Waleed Dandan, Design: Nusrat Khan, Data Collection and/or Processing: Nusrat Khan, Suha Hadi, Noura Al Hassani, Waleed Dandan, Analysis and/or Interpretation: Suha Hadi, Noura Al Hassani, Literature Research: Nusrat Khan, Suha Hadi, Noura Al Hassani, Writing: Nusrat Khan, Waleed Dandan.

Financial Disclosure: The authors declared that this study has received no financial support.

## Figures and Tables

**Table 1:**
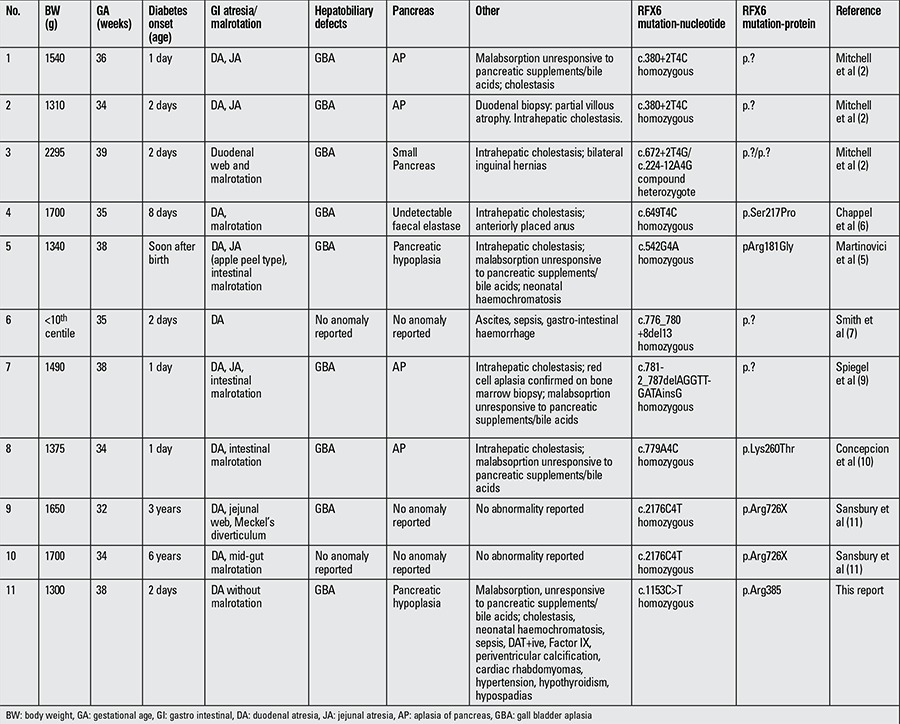
Reported cases of Mitchell-Riley syndrome
